# Push–Push Electrothermal MEMS Actuators with Si-to-Si Contact for DC Power Switching Applications

**DOI:** 10.3390/mi16090977

**Published:** 2025-08-26

**Authors:** Abdurrashid Hassan Shuaibu, Almur A. S. Rabih, Yves Blaquière, Frederic Nabki

**Affiliations:** Department of Electrical Engineering, École de Technologie Supérieure, Université du Québec, Montréal, QC H3C 1K3, Canada

**Keywords:** MEMS, switch, Si-to-Si contact, electrothermal actuators

## Abstract

MEMS switches offer great advantages over solid-state and conventional electromechanical switches, including a compact size and high isolation. This paper presents a novel silicon-to-silicon (Si-to-Si) MEMS switch featuring two suspended actuated platforms for DC power switching applications. The proposed design uniquely incorporates dual suspended chevron actuators, enabling bidirectional actuation, enhancing force generation, and improving overall switching performance. Leveraging the robustness of silicon, this Si-to-Si contact switch aims to enhance the reliability of MEMS-based DC power switches. Testing of a fabricated device in the PiezoMUMPs process demonstrated that a 2 μm initial contact gap closes at 1.1 *V*_DC_, with a total actuation power of 246 mW. The switch exhibits a linear voltage–current response up to 5 mA of switching current and achieves a minimum contact resistance of ~294 ± 2 Ω, one of the lowest reported for Si-to-Si contacts. This low contact resistance is attributed to the suspended contact platforms, which mitigate misalignment. The measured response time was 4 ms for turn-on and 2.5 ms for turn-off. This switch withstood a breakdown voltage of up to 376 V across the 2 µm contact gap. Moreover, the 200 nm thick oxide layer separating the actuation and signal lines exhibited breakdown at 183 V. These findings highlight the potential of the switch for high-voltage applications and pave the way for further enhancements to improve its reliability in harsh environments.

## 1. Introduction

Microelectromechanical systems (MEMSs) can sense and control various physical, optical, or chemical quantities, such as acceleration, radiation, or fluid flow [1]. Due to their high isolation, linearity, low insertion loss, and low power consumption, MEMS switches have garnered interest in recent years [2]. They are promising candidates to replace conventional electronic switches [3], which suffer from off-state leakage currents, limited linearity, and power efficiency issues [4]. Additionally, typical electromechanical switches tend to be bulky.

The long-term reliability of MEMS switches is dictated largely by the choice of contact material. Among the various options, highly doped silicon-to-silicon (Si-to-Si) ohmic contacts stand out for their high dielectric breakdown strength and mechanical robustness. Nevertheless, minimizing contact resistance in these devices remains challenging.

Work in [5] employed two heavily n-type doped single-crystal Si wafers (0.01–0.02 Ω·cm) fabricated with an in-house silicon-on-insulator (SOI) process. Prior to wafer bonding, both contact surfaces received a 400 nm metal overcoat, yielding a vertical inertial switch with an average on-state resistance of ~1 kΩ. A subsequent study [6] reported a vacuum-encapsulated Si-to-Si microswitch (SOI, 0.005–0.02 Ω·cm) whose resistance remained in the tens of kilohms despite identical substrate doping. In [7], an SOI switch driven by combined electrothermal and electrostatic actuation achieved 1.5 Ω by adding a layered metallization stack (50 nm chromium, 600 nm gold, and 1 µm copper) to the contacts. Other Si-to-Si implementations—for example, the nano-cantilever RF switch in [8] and the electrostatically actuated RF ohmic switch in [9], did not disclose measured contact resistance.

These results highlight a recurring trade-off: metal coatings, such as gold, consistently lower contact resistance thanks to their excellent conductivity and chemical inertness, yet their intrinsic softness accelerates wear and promotes stiction, limiting switch lifetime [10]. Hybrid pairs (e.g., gold-to-platinum or gold-to-iridium) mitigate adhesion but degrade over time owing to carbonaceous build-up, whereas silver-to-silver contacts are vulnerable to corrosion. Hermetic sealing has, therefore, been proposed to shield interfaces from environmental contaminants [2]. More refractory metals, including ruthenium and platinum, offer superior resistance to tribo-chemical contamination and may extend switch endurance compared to gold, albeit with added complexity and cost [10].

Against this backdrop, work has focused on engineering the contact interface so that Si-to-Si topologies can deliver relatively low on-state resistance without sacrificing their inherent advantages, an objective that motivates the present study. Electrothermal actuation was selected for its ability to supply the large forces required for low-resistance contacts. Illustrative precedents include the shape memory alloy (SMA)-driven switch with carbon nanotube (CNT) contacts reported in [11], providing high force and large displacement, and the chevron-type electrothermal–parallel-plate electrostatic hybrid that achieved 1 W power handling over 10 million cycles in [7].

In our prior work [12], a DC MEMS switch based on chevron-type electrothermal actuators was reported. It was observed that, upon contact between the moving doped silicon platform and the fixed doped silicon electrode, an out-of-plane misalignment of approximately 100 nm remained, leading to an increase in measured contact resistance. Reducing this misalignment is important, as it directly affects the doped contact surface area and, consequently, the switch’s electrical performance.

Accordingly, this paper proposes a novel architecture with a Si-to-Si ohmic contact MEMS switch in which both contact surfaces are suspended to mitigate out-of-plane misalignment post-actuation, rendering the structure more resilient to post-fabrication intrinsic stresses. The ability to separately actuate the two contact surfaces makes it easy to control and eliminate the out-of-plane misalignment of the two surfaces. A study with finite element analysis (FEA) simulations and experiments with fabricated designs demonstrates its out-of-plane misalignment control capability, with fairly fast electrothermal actuators with only a few milliseconds of response time. That study also shows that the Si–Si contact interface minimizes contact resistance while providing an excellent dielectric breakdown strength, thereby enabling long-term reliability without protective sealing. This design, made with standard MEMS processes, supports post-fabrication metal deposition to reduce switch contact resistivity while preserving dimensional accuracy, making it adaptable to diverse integration requirements. Together, these innovations establish a reliable, mechanically robust platform that is resistant to wear and contamination and viable for DC power switching applications.

## 2. Materials and Methods

### 2.1. Design

**Figure 1:**
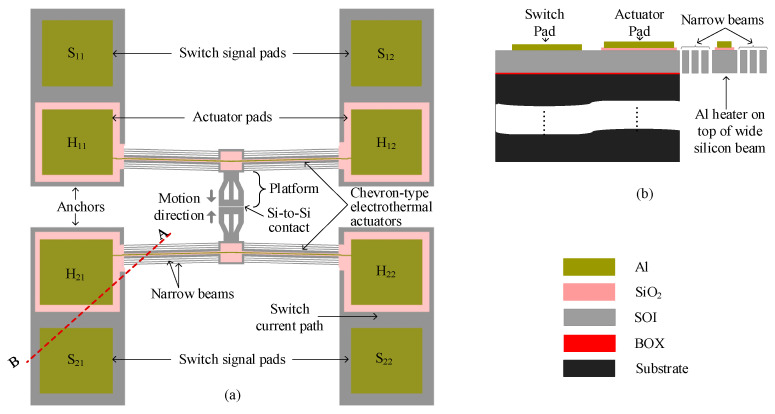
(**a**) Schematic of Si-to-Si contact DC power MEMS switch and (**b**) cross-sections A–B, showing the suspended and anchored parts.

A schematic of the Si-to-Si contact DC MEMS switch is shown in Figure 1a. The design features twin chevron-type push–push electrothermal actuators, symmetrically anchored to the substrate at both ends and connected to a suspended central platform. These actuators drive the platform, enabling a contact to be formed between the signal pad pairs.

Each actuator consists of a multilayer stack of aluminum (Al) and silicon dioxide (SiO_2_) on a wide SOI beam. The aluminum layer functions as a microheater, while the SiO_2_ layer provides electrical isolation between the heater and the signal path, preventing interference with the switched signal.

The chevron actuator comprises a symmetric array of slanted beams at an angle, β, which expand laterally when Joule heating is applied. As current flows through the microheater pads, thermal expansion of the beams pushes the central platform inward from both sides, closing the initial 2 μm gap between the switch signal pads (S_21_ and S_22_ to S_11_ and S_12_) and establishing electrical contact, as illustrated in Figure 1a. The post-switch closure current flow is indicated by the light blue path in the figure.

Figure 1b shows a cross-section in which the SiO_2_ layer electrically isolates the switch pads from the actuator pads. On either side of the main SOI beams, parallel 3-beam arrays provide the restoring force that reopens the switch when actuation ceases, conduct heat away from the contact region to limit temperature rise, and improve the overall mechanical stability of the structure.

To meet fabrication constraints, the minimum width of the aluminum heater layer is set at 5 μm, while the SiO_2_ and SOI layers have respective allowable widths of 11 μm and 32 μm, as summarized in Table 1, in which all design parameters are listed. The layer enclosure rules, dictated by the process design, require the SiO_2_ layer to laterally enclose the Al-Cr heater stack by at least 5 μm, while the silicon structural layer must enclose the oxide by a minimum of 3 μm [13]. These were the minimum allowed; however, larger values were encouraged to ensure the good functionality of the design. As such, this constraint introduces a notable design trade-off, increasing the aspect ratio of the multilayer beams such that the silicon layer thickness (10 μm) becomes more than three times smaller than the beam width (32 μm). As a result, the structure becomes more prone to out-of-plane deflection during actuation. However, the proposed design mitigates this challenge by suspending both contact surfaces and employing symmetric, simultaneous actuation. This configuration ensures that any residual out-of-plane deflection is effectively canceled out, preserving alignment between contacts and maintaining consistent low-resistance switching performance.

### 2.2. Finite Element Analysis (FEA) Simulations

FEA simulations were performed using the CoventorMP software 11.1 (Coventor, Raleigh, NC, USA) to evaluate the performance of the proposed design, implemented in the PiezoMUMPs process (Science Cooperation, Inc., Alameda, CA, USA) [13]. The same process was used for fabrication, with details provided later. The FEA simulation in CoventorMP follows four main steps. First, the material database is defined by specifying the properties of the materials used, including Young’s modulus, density, residual stress, coefficient of thermal expansion, thermal conductivity, and specific heat capacity, as these parameters influence actuator displacement. Second, the process definition is established, where layers are arranged according to the fabrication sequence, incorporating deposition and etching cycles to define layer thicknesses. Third, the layout design is created, where each layer is drawn in 2D to define its dimensions, while the thicknesses are set in the process definition. The 3D model is then meshed, and the necessary boundary conditions are assigned. Finally, an appropriate solver is selected to simulate the design.

**Figure 2:**
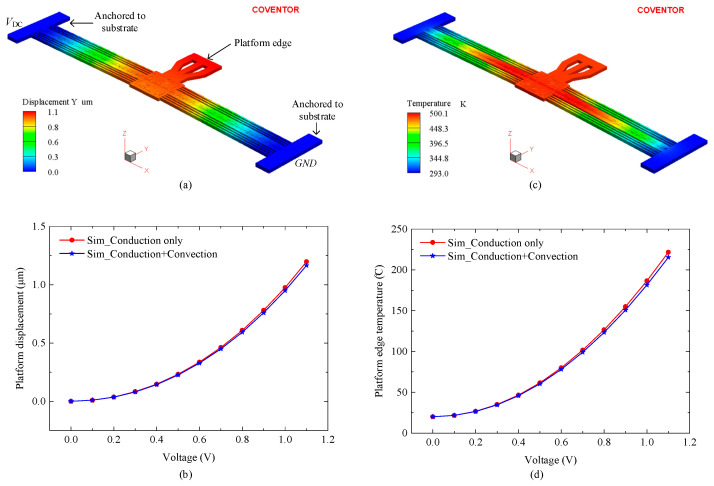
FEA simulation results showing (**a**) displacement and (**c**) temperature profiles at 1.1 V actuation. (**b**,**d**) Platform edge displacement and temperature, respectively, versus the actuation voltage.

For this switch design, an electro-thermo-mechanical physics solver was employed to analyze the actuator displacement due to Joule heating generated by applying a DC actuation voltage (*V*_DC_) across the chevron beam terminals, as shown in Figure 2a. The model was fixed to the substrate at anchoring points, which were set at room temperature. Two simulation cases were considered: the first case studied the actuator response under conductive heat transfer only, while the second case included both conduction and convection mechanisms. The electro-thermo-mechanical properties affecting the switch’s performance are summarized in Table 2. These parameters are predefined in the PiezoMUMPs process design kit (PDK); however, slight deviations may exist in the final fabricated devices.

As the switch consists of two identical suspended structures that move against each other, only one structure was simulated to evaluate performance, as illustrated in Figure 2a. To close the initial 2 μm contact gap, each microheater needed to generate 1 μm of displacement. Figure 2b presents the platform displacement versus actuation voltage, where the voltage was increased from 0 V to 1.1 V in 0.1 V increments. The displacement increased with actuation voltage, reaching 1.1 μm at 1.1 V, consistent with Figure 2a. The inclusion of convection in the thermal model had a negligible effect on actuator performance, resulting in displacement discrepancies of less than 5%. At 1.1 V, the difference in displacement between the conduction-only and conduction-plus convection models was 2.8%. This result is consistent with findings reported in [14], which indicates that, for the static characterization of electrothermal actuators, heat loss through convection is negligible compared to conduction.

Temperature plays an important role in the long-term reliability of MEMS switches, as excessive heating accelerates mechanical degradation and failure [15]. Figure 2c shows a maximum temperature of 501 K (227 °C) recorded at a 1.1 V actuation voltage. Figure 2d illustrates the temperature variation with actuation voltage, demonstrating a direct correlation between increased heating voltage and rising temperature.

### 2.3. Microfabrication and SEM Characterization

**Figure 3:**
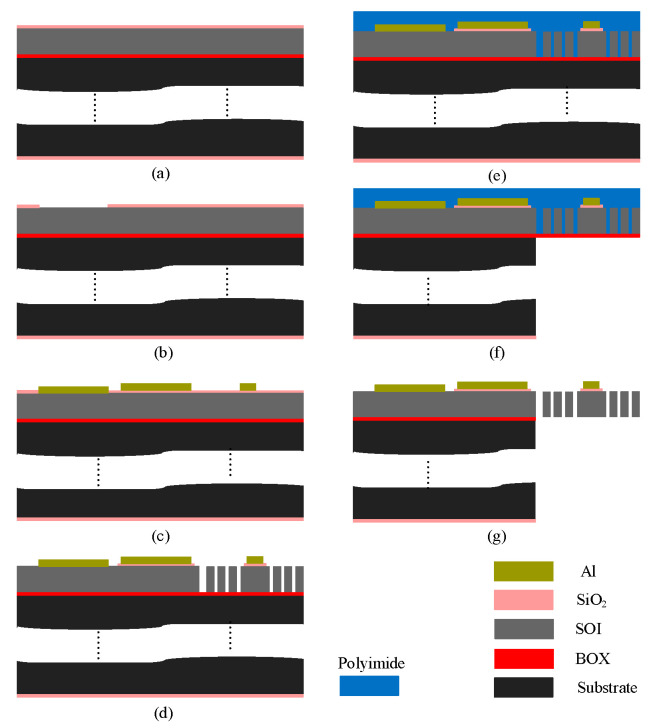
Fabrication process steps: (**a**) thermal oxide growth, (**b**) oxide patterning, (**c**) metal deposition and patterning, (**d**) front-side SOI etching, (**e**) front-side polymer coating, (**f**) backside handle layer etching, and (**g**) release of the MEMS structure.

The push–push MEMS switch was fabricated using the PiezoMUMPs process [13], illustrated in Figure 3. This fabrication process employs a 6-inch, n-type (100) silicon wafer with a 400 μm thick handle substrate, a 1 μm buried oxide (BOX) layer for insulation, and a 10 μm thick SOI device layer. The fabrication of the electrothermal-based MEMS switch required four masks and began with n-type surface doping of the SOI layer. This was achieved by depositing a phosphosilicate glass (PSG) layer, followed by annealing at 1050 °C for 1 h in an argon atmosphere. The PSG layer was then removed, and a 200 nm thermal oxide was grown on both wafer surfaces to serve as a pad oxide electrical insolation layer, as shown in Figure 3a.

The first photolithography step involved coating the wafer’s front side with a photoresist, followed by patterning using a padoxide mask. The thermally grown oxide layer was etched using reactive ion etching (RIE) to expose windows down to the SOI layer, facilitating metal deposition for direct electrical contact with the silicon switch contacts (Figure 3b). The wafer was again coated with photoresist and patterned using a padmetal mask before depositing a 20 nm chromium (Cr) adhesion layer and a 1 μm Al layer via E-beam evaporation. The metal patterning was completed through a lift-off process to form the microheater and contact pads, as illustrated in Figure 3c.

Then, the metal-patterned wafer was coated with photoresist, and the SOI mask was used to define the silicon device layer. First, the remaining front-side oxide layer was etched using RIE, followed by deep reactive ion etching (DRIE) of the silicon device layer, using the BOX layer as an etch stop mask, as depicted in Figure 3d. After DRIE, the front side was coated with polyimide as a protective layer in preparation for backside etching (Figure 3e). The wafer was then flipped, and the backside was coated with photoresist, followed by patterning using the trench mask. The backside thermal oxide was etched using RIE, and the handle layer was etched using DRIE, stopping at the BOX layer (Figure 3f). Once etching was completed, the photoresist was removed, and a wet oxide etch was used to dissolve the BOX layer. Finally, the photoresist and oxide layers were stripped, and a dry etch of the polyimide layer was performed to release the final structure, as shown in Figure 3g.

It is worth noting that despite fabricating the devices using the same materials described in Table 2, the parameter values of these materials are based on the PDK available with the simulation tool (CoventorWare). However, the exact parameter values used by the foundry to fabricate the devices are not accessible to the users.

**Figure 4:**
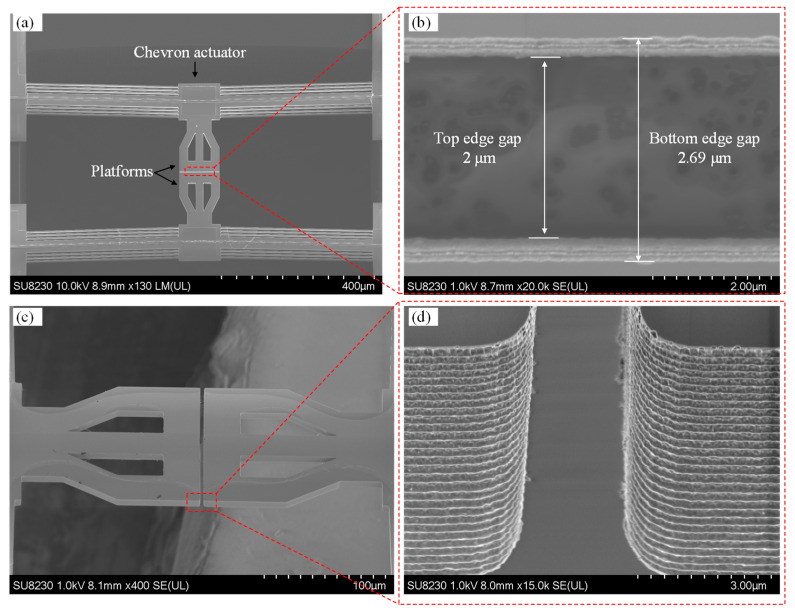
SEM micrographs of the MEMS switch: (**a**) top view showing the chevron actuators, platforms, and pads; (**b**) backside image displaying the front and backside gap edges; (**c**) 45° tilted view of the platforms; and (**d**) close-up view of the contact gap.

Prior to experimental testing, the fabricated devices were inspected using a SEM (model SU8200, Hitachi High-Technologies, Corporation, Tokyo, Japan), with micrographs presented in Figure 4. Figure 4a shows a top-down view of the fully released MEMS device. Visible in this image are the chevron electrothermal actuators, suspended contact platforms, and bond pads (both actuation and signal pads). The image confirms that the structural release was successful, with no residual material blocking the motion paths of the actuators or the central platform. The symmetry of the actuator arms and the alignment of the central platform suggest proper fabrication, which is essential for reliable in-plane actuation and switch operation. Figure 4b provides a zoomed-in cross-sectional view of the contact region, focusing on the air gap between the mating contact surfaces at the top and bottom edges of the silicon-on-insulator (SOI) layer. The top edge gap closely matches the designed 2 μm, while the bottom edge gap is larger, measured at 2.69 μm. This non-uniformity in the vertical gap is attributed to the slight tapering of the sidewalls caused by limitations in the deep reactive ion etching (DRIE) process. Specifically, the measured difference of ~690 nm corresponds to an etch angle of approximately 88.08°, deviating from the ideal 90°. This taper introduces a bottom air gap that may not fully close upon actuation, possibly affecting contact resistance and reliability. Figure 4c shows a tilted perspective view of the MEMS switch, revealing the three-dimensional geometry of the actuators and the suspended structure. This view helps to visualize the vertical spacing between layers and the depth of the etched regions. The figure also highlights the symmetry and alignment of the chevron actuators that drive the central platform from both sides, critical for minimizing out-of-plane displacement. Figure 4d offers a high-magnification close-up of the contact surfaces. The striated or rippled texture observed on these surfaces is a characteristic artifact of the DRIE process, which alternates between etching and passivation cycles (commonly referred to as the Bosch process). These surface irregularities can influence both the contact resistance and the mechanical adhesion at the interface. Rougher surfaces may reduce the real contact area or create unintended anchoring points, potentially increasing stiction or variability in electrical performance. In summary, this figure confirms the successful fabrication of the device while also revealing process-induced imperfections such as sidewall tapering and surface roughness. These aspects are critical, as they directly influence the electrical contact quality, out-of-plane misalignment, and overall reliability of the MEMS switch.

## 3. Experimental Setup and Results

### 3.1. Test Setups

**Figure 5:**
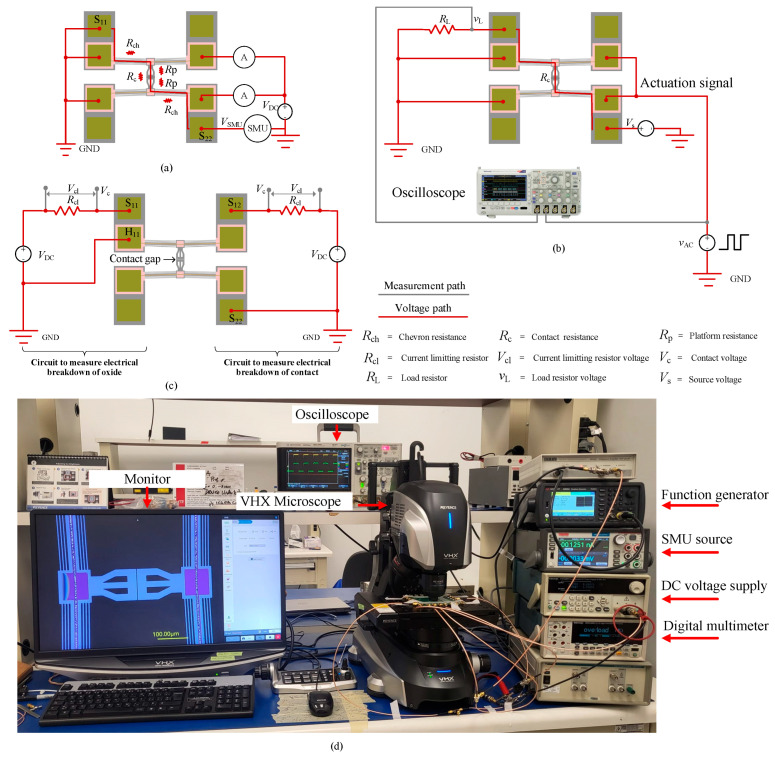
Experimental setups: (**a**) measurement setup for platform displacement and contact resistance, (**b**) setup for switch response time measurement, (**c**) setups for breakdown voltage measurement (right-side and left-side circuits were measured separately), and (**d**) photograph of the equipment used for these setups.

To characterize the MEMS switch, four types of experiments were conducted. The first experiment measured the platform displacement as a function of actuation voltage to determine the precise voltage required to close the initial 2 μm contact gap and activate the switch. As described in Section 2, the switch consists of two push–push actuators, meaning each actuator’s platform must travel half the total displacement to achieve switch closure. The test setup, shown in Figure 5a, included a DC voltage source supplying an actuation voltage (*V*_DC_) ramped from 0 V to 1.1 V in 0.1 V increments. Ammeter readings were taken to measure the current and estimate the power consumption of each actuator. A VHX microscope (Keyence, Itasca, IL, USA) was used to observe and monitor platform movements during actuation.

Once the closure voltage was identified, the second experiment measured Si-to-Si switch contact resistance by analyzing the current–voltage (I-V) characteristics. A Keithley source measure unit (SMU) (Tektronix, Beaverton, OR, USA) was connected through the contact pads (S_11_ and S_22_), as illustrated in Figure 5a. When the switch was closed, the SMU applied a small test current (*I*_SMU_) and measured the resulting voltage (*V*_SMU_) to generate the I-V curve. The switch contact resistance at each point was then calculated using Ohm’s law. Measurements were conducted at *I*_SMU_ = 1 mA, 2 mA, 3 mA, 4 mA, and 5 mA, with the SMU automatically adjusting the applied voltage based on the contact resistance value. Currents exceeding 5 mA caused rapid degradation of the aluminum heater that actuated the switch.

The third experiment evaluated the switch response time, using the test setup depicted in Figure 5b. A square wave actuation signal (*v*_AC_) with a 50% duty cycle, 1 Hz frequency, and 2.14 V_pp_ (peak-to-peak) with a 500 mV DC offset was applied to the microheater terminals via a Keysight 33600A Waveform Generator (Santa Rosa, CA, USA). A 600 kΩ external load resistor (*R*_L_) was connected in series with the switch resistance (*R*_SW_). A 5 V DC source (*V*_S_) was applied across the resistors to allow for current flow upon switch closure. To measure the switch-on time (*T*_on_) and switch-off time (*T*_off_), a DSO-X 3034A oscilloscope (Agilent Technologies, Santa Clara, CA, USA) was used. One oscilloscope channel captured the actuating square wave signal, while another measured the voltage drop (*v*_L_) across *R*_L_ upon switch closure. The *T*_on_ (and *T*_off_) was determined by measuring the time delay of *v*_L_ at the rising (and falling) edge of the actuating signal, *v*_AC_.

The fourth measurement aimed to determine the breakdown voltages of both the contact gap and the oxide layer, which isolate the actuation and signal lines. This measurement allowed for the evaluation of the integrity of the switch from two perspectives. Firstly, the breakdown voltage of the contact gap indicates the switch’s ability to withstand high-voltage surges, which are common in harsh environments. If the breakdown voltage is too low, the switch may unintentionally turn on, even when the actuators are inactive, potentially leading to device failure. Secondly, the oxide layer is responsible for electrically isolating the actuators from the signal lines. To ensure proper functionality, this layer must possess adequate dielectric strength and thickness to prevent dielectric breakdown and signal–actuation crossover. As illustrated on the right side of Figure 5c, the contact gap breakdown voltage was measured by connecting a current-limiting resistor (*R*_cl_) in series with the switch contact and applying a DC voltage (*V*_DC_) across S_12_ and S_22_, with *R*_cl_ >> R_SW_ (in a closed state). The voltage across the RC series circuit was monitored, where *V*_cl_ represents the voltage drop across the resistor, and *V*_C_ corresponds to the voltage drop across the contact.

Initially, with the contact open, no current flowed, causing *V*_CL_ to drop to zero, while *V*_C_ remained equal to the applied *V*_DC_. As *V*_DC_ increased, the contact gap progressively reduced until the breakdown voltage was reached, leading to an electrical breakdown. At this point, a sudden increase in current caused nearly the entire *V*_DC_ to drop across *R*_CL_, making *V*_CL_ ≈ *V*_DC_, while *V*_C_ approached zero. This same procedure was applied to measure the oxide layer breakdown voltage (on the left side of Figure 5c), where *V*_DC_ was applied across the contact pad (S_11_) and the heater pad (H_11_). Note that the breakdown voltages of the switch contact and the oxide layer were measured using separate devices to eliminate any potential interference of one over the other.

### 3.2. Results

**Figure 6:**
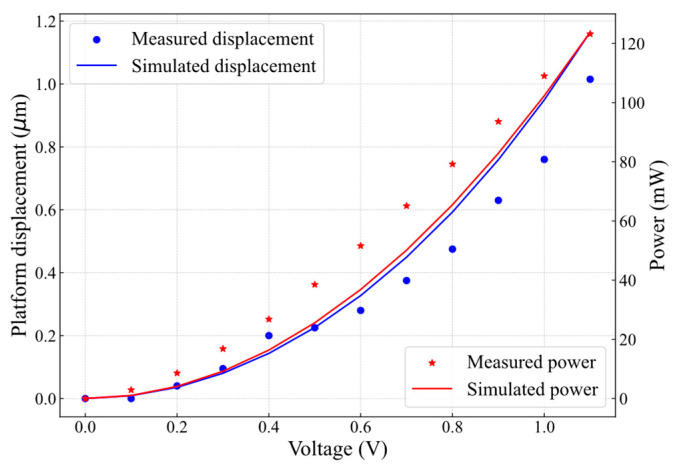
Measured and simulated platform displacement and actuator power as a function of microheater actuation voltage.

For displacement measurement, due to the symmetry of the actuators and platforms, and since the same voltage is applied to both actuators simultaneously, the total measured displacement is divided by two to obtain the displacement of each actuator. Figure 6 presents the measured and simulated platform displacement and actuator power versus actuation voltage. The displacement increased with applied voltage, reaching 1.02 μm at 1.1 V (123.2 mW per actuator) in the experimental results, while simulations predicted 1.13 μm, leading to a ~10% discrepancy. This variation is attributed to differences between the material properties used in fabrication and those assumed in the simulation model, as well as to geometry variations due to fabrication precision. Nevertheless, both curves exhibit a similar trend, validating the developed simulation model.

**Figure 7:**
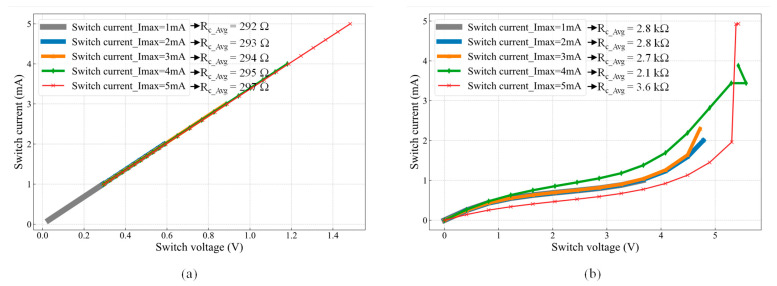
I-V characteristics of the MEMS switch: (**a**) first cycle showing a Si-to-Si contact resistance (*R*_c_) of ~294 Ω and (**b**) second cycle exhibiting an increased *R*_c_ of ~3 kΩ.

The switch contact resistance, *R*_SW_, was measured using the same setup shown in Figure 5a, where the switch was actuated by applying *V*_DC_ = 1.1 V. Since the SMU was connected between contact pads (S_11_ and S_22_) (as shown in Figure 5a), the measured resistance comprises resistances of the two chevron actuators (*R*_ch_), the two platforms (*R*_p_), and the contact resistance (*R*_c_). Given the relatively high conductivity of the SOI layer (2.0 × 10^9^ pS/μm), the contributions of *R*_ch_ and *R*_p_ are negligible (<2%) compared to *R*_c_. Therefore, throughout this study, the measured switch resistance, *R*_SW_, is assumed to correspond to the contact resistance (*R*_SW_ ≈ *R*_c_). Figure 7a shows the first I-V measurement cycle, where the tested currents (*I*_SMU_ = 1 mA, 2 mA, 3 mA, 4 mA, and 5 mA) exhibit a linear relationship with the corresponding voltage drop.

The contact resistance (*R*_c_) for each test current was calculated using Ohm’s law. The average contact resistance (*R*_c_Avg_), as shown in Figure 7a, slightly increased with higher test currents due to Joule heating. Specifically, *R*_c_Avg_ = 292 Ω for *I*_SMU_ = 1 mA, increasing to 297 Ω for *I*_SMU_ = 5 mA. Further increasing the test current beyond 5 mA resulted in a non-ohmic I-V curve, likely due to a temperature increase, as discussed in [2]. While the first I-V cycle exhibited an ohmic response, reopening and reclosing the switch led to a non-ohmic behavior and increased contact resistance, as shown in Figure 7b.

The observed degradation is attributed to excessive localized heating in the microheaters during actuation. Repeated Joule heating elevates the microheater temperature beyond its thermal stability limit, leading to material degradation, such as metal diffusion, increased resistivity, reflow, and potential oxidation of the heater material. Additionally, thermal stress caused by mismatched coefficients of thermal expansion (CTE) between the heater layers and the silicon substrate can induce mechanical fatigue and delamination over multiple actuation cycles. The actuator degradation could also be attributed to the reduction reaction of the aluminum–oxide–silicon, as explained in [16]. As a result, *R*_c_Avg_ increased significantly, reaching values between 2.1 kΩ and 3.6 kΩ, confirming irreversible microheater damage and a degraded electrical response in subsequent switching cycles, where the contact resistance values increased to more than 10 kΩ.

**Figure 8:**
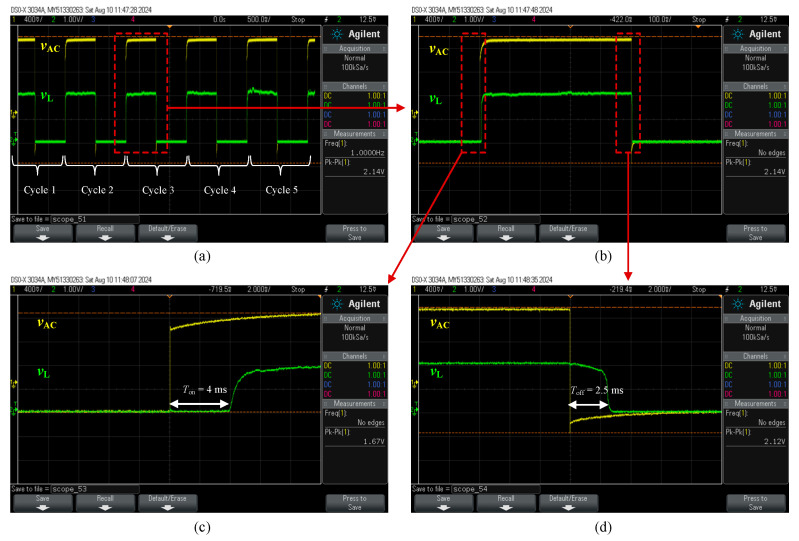
Response time of the switch: (**a**) over five measurement cycles, (**b**) zoomed-in view of cycle 3, (**c**) measured switch-on time (*T*_on_), and (**d**) measured switch-off time (*T*_off_).

The mechanical response time of the switch was measured using the setup in Figure 5b, with results shown in Figure 8. The modulated voltage drop (*v*_L_) across the load resistor (*R*_L_) was recorded over five switching cycles, as presented in Figure 8a. The switch-on time (*T*_on_), extracted from the rising edge of the third cycle’s positive peak in Figure 8b, was measured as 4 ms, as shown in Figure 8c. Notably, all cycles exhibited consistent response times. The switch-off time (*T*_off_), determined from the falling edge of *v*_L_, was measured as 2.5 ms, as shown in Figure 8d. The shorter *T*_off_ is attributed to the sharp drop of the actuation signal (*v*_AC_) on the falling edge compared to the slower rising edge, as observed in Figure 8. Similar behavior has been reported in [7,17] for electrothermal Si-to-Si contact switches, where the longer *T*_on_ results from the additional time required for the actuators to close the initial contact gap, whereas *T*_off_ is governed by the immediate disconnection of the contact surfaces [7]. It is important to note that the mechanical response time of the switch is influenced by the thermal response time of the actuators, which is governed by the thermal diffusion rate within the actuator structure [18]. Consequently, the thermal response time is expected to be shorter than the total mechanical actuation time.

A distinction should be made between the measurement conditions in Figure 7 and Figure 8. In Figure 7, the measured parameter was the contact resistance, obtained by energizing the actuators with 1.1 *V*_DC_, corresponding to 246 mW, and applying a maximum sensing current of 5 mA using an SMU. In contrast, for the response time measurement shown in Figure 8, an AC signal of 2.14 V_pp_ was applied, with an AC power lower than the DC power used in the contact resistance test, yet still sufficient to establish contact. Under these conditions, the contact resistance was in the MΩ range, and the corresponding testing current was in the μA range. Furthermore, the use of an AC signal with a 50% duty cycle helped minimize thermal stress, thereby extending the actuator’s lifetime. As a result, no heater degradation was observed during the response time measurements, and the switching cycles remained repeatable.

**Figure 9:**
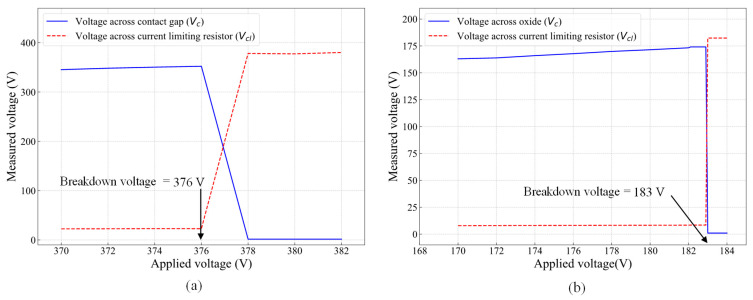
High-voltage handling showing (**a**) breakdown voltage of the 2 μm wide contact gap and (**b**) breakdown voltage of the 200 nm thick oxide layer.

The breakdown voltages of the 2 μm wide contact gap and 200 nm thick oxide layer that separate the actuation and signal lines were measured using the setup shown in Figure 5c. The results are shown in Figure 9. The applied DC voltage, *V*_DC_, was ramped from 0 to 382 V in 1 V increments per second while monitoring the voltage across the contact gap, *V*_c_, and the voltage drop across the current limiting resistor, *V*_cl_. In Figure 9a, the breakdown occurred at 376 V, when the voltage across the contact gap dropped sharply from about 350 V to 1.7 V, while the voltage drops across the resistor increased sharply from about 22 V to 376 V. The same procedure was repeated to determine the breakdown voltage of the oxide layer, which was found to occur at 183 V, as shown in Figure 9b. These measurements were conducted at atmospheric pressure and room temperature. Thus, changing the ambient pressure or temperature is expected to influence the measurements, as the initial contact gap will change with temperature changes, and electric breakdown across the gap is a function of air pressure.

## 4. Discussion

Notwithstanding the heater damage issue observed, the novel electrothermal-based Si-to-Si contact MEMS switch presented in this manuscript was developed for high-DC power switching applications. The experimental results demonstrated that an ohmic contact resistance as low as 292 Ω and a response time of 4 ms were achieved at 1.1 *V*_DC_, with a total actuation power of 246 mW for the two actuators. To the best of the authors’ knowledge, this represents the lowest reported ohmic resistance for a Si-to-Si contact composed entirely of single-crystal silicon teched with the Bosch DRIE process without metal deposition on the contact parts. Table 3 compares this work to state-of-the-art Si-to-Si contact ohmic switches. In [7], a 1.5 Ω contact resistance and 70 ms response time were reported for an SOI-based switch utilizing electrothermal and electrostatic actuation techniques, with a thick metallic deposition (50 nm chromium, 600 nm gold, and 1 μm copper) on the contact surfaces. In [17], a 100 Ω contact resistance and a 0.43 ms response time were demonstrated. However, a cryogenic DRIE process that produced roughness-free sidewalls on a 5 Ω·cm SOI wafer was used instead of Bosch DRIE. In [5], despite 400 nm thick metal deposition on the silicon contacts, an average contact resistance of ~1 kΩ and a response time of 0.17 ms were reported. In [6], a Si-to-Si contact microswitch was vacuum-encapsulated and tested, yet it exhibited a contact resistance in the tens of kilo-ohms range.

Despite achieving a low Si-to-Si contact resistance of 292 Ω in the first measurement cycle without the use of metal coating, the results were not repeatable, with the contact resistance increasing to the kilo-ohm range in the second cycle. In addition to heater degradation, which reduces the available contact force and thus compromises the quality of the electrical interface, several other contributing factors were identified. These include surface roughness at the contact interface, out-of-plane misalignment between the contact platforms, and non-uniform doping profiles introduced during fabrication.

First, Figure 4d reveals noticeable surface roughness at the contact interface, attributed to scallops formed during the DRIE process. This roughness results from alternating etching and passivation cycles, where silicon etching is followed by fluorocarbon polymer deposition to protect the sidewalls. An alternative cryogenic DRIE process can mitigate this issue, as it replaces fluorocarbon polymer coatings with oxide/fluoride-blocking layers, allowing for smoother etching while maintaining high aspect ratios [19], as illustrated in the study presented in [17], where as low as 100 Ω with a good repeatability of only 10% variation in contact resistance over 5 million switching cycles was reported. This indicates that improving the sidewall profile could result in lower and more repeatable contact resistance values.

**Figure 10:**
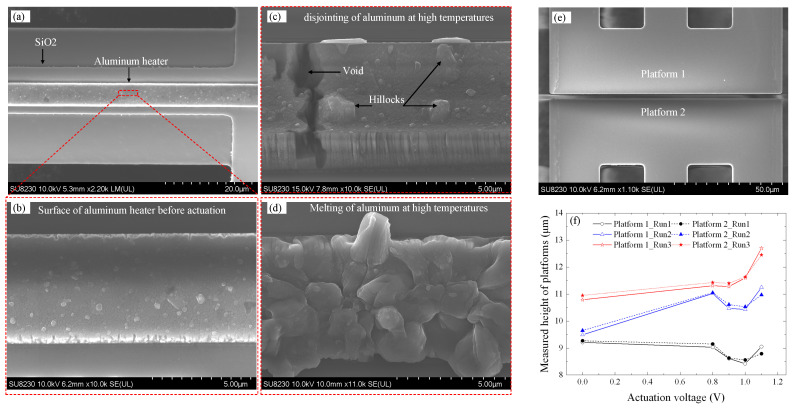
(**a**) Aluminum heater before activation; (**b**) zoomed-in view of the heater; (**c**) [20] and (**d**) heater degradation due to high-temperature operation, showing disjointing and melting; (**e**) two platforms forming the contact; and (**f**) platform height measurements relative to a fixed reference on the die surface at different actuation voltages over three run cycles.

Second, as was previously discussed, the device suffered heater damage after the first actuation. A portion of the heater is shown in Figure 10a, with a close-up in Figure 10b. To close the contact gap and achieve sufficient contact force, each actuator required a high current of ~112 mA, resulting in localized temperatures exceeding 200 °C, as confirmed by simulation. Over time, excessive Joule heating led to heater degradation, as evidenced by the formation of hillocks and voids in Figure 10c. Prolonged operation caused further melting and eventual disjointing of the aluminum heater layer, as shown in Figure 10d, which significantly impaired current conduction and ultimately resulted in complete heater failure. At high temperatures, disjointing of aluminum leads to mechanical degradation, increased electrical resistance, and interfacial failure due to grain boundary weakening, thermal expansion mismatch, and atomic diffusion. These phenomena compromise both the structural and electrical integrity of the heater, resulting in poor heat dissipation, circuit malfunction, and reduced reliability, particularly under prolonged thermal and electrical stress. The deterioration of the heater layer contributed to non-uniform thermal expansion, leading to mechanical instability in the actuator. Consequently, out-of-plane misalignment of the contact platforms, shown in Figure 10e, increased progressively over successive actuation cycles, as plotted in Figure 10f for three test runs. This out-of-plane displacement was found to be permanent, with platform heights increasing after each run, even in the absence of applied voltage. Measured relative to a fixed die reference, the heights at 1.1 V rose from ~9 μm in Run 1 to ~11 μm in Run 2 and beyond 12 μm in Run 3. While both platforms exhibited similar vertical drift, the relative misalignment between them was initially below 100 nm and then exceeded 300 nm at 1.1 V. This increased misalignment is attributed to overdriving-induced thermal stress, coupled with the progressive structural warping associated with aluminum layer breakdown. Therefore, by replacing or improving the heater layer, the possibility to independently actuate each platform/contact surface makes it easy to control and eliminate the out-of-plane misalignment of the two surfaces by applying slightly different power levels to each corresponding actuator.

Additionally, contact resistance was further exacerbated by etch angle variations at the interface. As discussed in [19,20], optimizing the sidewall etch angle toward 90° would improve contact conformity, reduce overdrive-induced misalignment, enlarge the effective contact area, and further lower the on-state contact resistance.

It is important to note that, as the Si-to-Si contact was formed via the etching of a surface-doped SOI wafer, the doping concentration was highest at the top surface and gradually decreased toward the bottom of the Si layer, requiring good alignment between each platform. As such, with significant misalignment, when force was continuously applied to improve contact between the highly doped regions, adjacent less-doped regions of the sidewalls could have made contact, leading to the increased resistance observed in subsequent test cycles.

**Figure 11:**
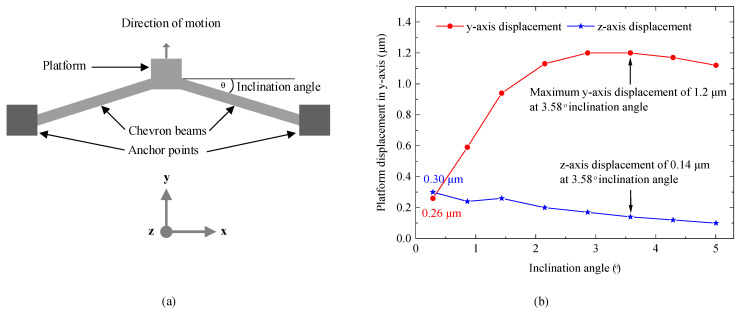
(**a**) Schematic of a chevron actuator and (**b**) optimization of the inclination angle, showing maximum *y*-axis displacement with minimal out-of-plane *z*-axis displacement at 3.58°.

To address the challenges observed in this design, a new push–push switch with optimized actuators was proposed. Since device dimensions were constrained by the fabrication process, the inclination angle of the chevron actuators was optimized. This angle is shown in Figure 11a, and it is important to optimize in order to minimize energy consumption, reduce contact temperature, and increase contact force, all of which enhance switch reliability. In this optimization of the actuator, the length, width, and thickness of the chevron beams were fixed, adhering to the PiezoMUMPs process constraints described in Section 2. The optimization process was conducted on a single chevron actuator from the switch model in Figure 1a. A constant voltage of 1 V was applied across the heater terminals, with the structure anchored at 20 °C. The *y*-axis (lateral) and *z*-axis (out-of-plane) displacements were monitored for inclination angles ranging from 0.3° to 5°, with a ~0.7° step size, with simulation results shown in Figure 11b. The electrothermal actuators exhibit bimorph behavior at small inclination angles (~0°), where out-of-plane displacement dominates over lateral motion. At 0.3°, the displacement on the *y*-axis was 0.26 μm, while the *z*-axis displacement was 0.30 μm. Increasing the angle enhanced the y-axis displacement while suppressing unwanted *z*-axis motion. The optimal inclination angle was identified as 3.58°, yielding a maximum *y*-axis displacement of 1.2 μm with minimal *z*-axis displacement (0.14 μm). Beyond this angle, displacements in both axes decreased.

At this optimized angle, the simulated contact temperature was ~158 °C, significantly lower than in the tested device in this work, which had an inclination angle of 1.43°, yielding a y-axis displacement of 0.94 μm and an undesirable *z*-axis displacement of 0.26 μm at 1 V. However, since the switch required 1.1 V for sufficient actuation, excessive Joule heating led to temperatures exceeding 200 °C in that device. With the optimized inclination angle, this optimized actuator is expected to make a second-generation switch mitigate the heater issue discovered in this work.

## 5. Conclusions

This paper presented a Si-to-Si ohmic contact MEMS switch designed for DC power applications. The switch consisted of two suspended platforms controlled by electrothermal actuators, separated by a 2 μm gap. Actuation forced the platforms into contact by closing this gap. Four types of experimental tests were conducted to evaluate displacement, contact resistance, response time, and voltage breakdown. For the first three measurements, actuation voltages ranging from 0 to 1.1 V were applied, with a maximum contact test current of 5 mA.

Compared to previously reported Si-to-Si contact switches, including those with metal-coated contacts, the proposed switch exhibited a low contact resistance of ~292 Ω, actuated at 1.1 V with a total power consumption of 246 mW. Dynamic testing results showed a switch-on time of 4 ms and a switch-off time of 2.5 ms. The switch also demonstrated high breakdown voltages of 376 V across the contact gap and 183 V across the oxide layer separating the actuation and signal lines.

Despite the limited repeatability of the contact resistance, attributed to factors such as heater degradation, FEA simulations of the optimized actuator design predict improved expected performance. These enhancements will be the focus of future work to render the design repeatable.

Looking ahead, future work will focus on implementing this metal coating step, refining the actuator structure, and improving the device’s endurance under repeated switching cycles. Additionally, the switch’s compact footprint, fast actuation speed, and high voltage tolerance make it well suited for power management in reliability-critical applications where low power consumption and rapid, non-volatile switching are essential.

## Figures and Tables

**Table 1:** Design parameters of the DC power MEMS switch.

Parameter	Value
Actuator length (μm)	400
Actuator width (μm)	
SOI	32
SiO_2_	11
Al	5
Narrow beam width (μm)	4
Contact gap (μm)	2
Footprint (mm^2^)	2.92

**Table 2:** Electro-thermo-mechanical parameters used in simulations.

Property	Value		
	Al	Si	SiO_2_
Young’s modulus (GPa)	57	170	70
Density (kg/μm^−3^)	19,300	2329	2150
Stress_X,Y (MPa)	50	15	0
TCE (1/K)	1.41 × 10^−5^	2.6 × 10^−6^	5.0 × 10^−7^
Thermal conductivity (pW/μmK)	2.97 × 10^8^	1.3 × 10^8^	1.4 × 10^6^
Specific heat (pJ/kgK)	1.29 × 10^14^	7.0 × 10^14^	1.0 × 10^15^
Electrical conductivity (pS/μm)	1.82 × 10^13^	2.0 × 10^9^	0

**Table 3:** State-of-the-art Si-to-Si MEMS ohmic contact switches.

Actuation Type	Contact Type	Contact Resistance	Response Time	Ref.
Electrothermal–electrostatic	Cu-coated Si-to-Si	1.5 Ω	70 ms	[7]
Electrothermal	Si-to-Si with Cryo DRIE	100	0.43 ms	[17]
Electrostatic	Metal-coated Si-to-Si	1 kΩ	0.17 ms	[5]
Electrostatic	Encapsulated Si-to-Si	35–45 kΩ	-	[6]
Chevron-type electrothermal	Si-to-Si with Bosch DRIE	292 Ω	4 ms	This work

## Data Availability

The original contributions presented in this study are included in the article; further inquiries can be directed to the corresponding author.
